# Application of a deep convolutional neural network in the diagnosis of neonatal ocular fundus hemorrhage

**DOI:** 10.1042/BSR20180497

**Published:** 2018-12-07

**Authors:** Binbin Wang, Li Xiao, Yang Liu, Jing Wang, Beihong Liu, Tengyan Li, Xu Ma, Yi Zhao

**Affiliations:** 1 Center for Genetics, National Research Institute for Family Planning, Beijing 100081, China; 2 Key Laboratory of Intelligent Information Processing, Advanced Computer Research Center, State Key Laboratory of Computer Architecture, Institute of Computing Technology, Chinese Academy of Sciences, Beijing 100190, China; 3 Department of Medical Genetics and Developmental Biology, School of Basic Medical Sciences, Capital Medical University, Beijing, China

**Keywords:** artificial intelligence, convolutional neural network, fundus images, retinal hemorrhage

## Abstract

There is a disparity between the increasing application of digital retinal imaging to neonatal ocular screening and slowly growing number of pediatric ophthalmologists. Assistant tools that can automatically detect ocular disorders may be needed. In present study, we develop a deep convolutional neural network (DCNN) for automated classification and grading of retinal hemorrhage. We used 48,996 digital fundus images from 3770 newborns with retinal hemorrhage of different severity (grade 1, 2 and 3) and normal controls from a large cross-sectional investigation in China. The DCNN was trained for automated grading of retinal hemorrhage (multiclass classification problem: hemorrhage-free and grades 1, 2 and 3) and then validated for its performance level. The DCNN yielded an accuracy of 97.85 to 99.96%, and the area under the receiver operating characteristic curve was 0.989–1.000 in the binary classification of neonatal retinal hemorrhage (i.e., one classification vs. the others). The overall accuracy with regard to the multiclass classification problem was 97.44%. This is the first study to show that a DCNN can detect and grade neonatal retinal hemorrhage at high performance levels. Artificial intelligence will play more positive roles in ocular healthcare of newborns and children.

## Introduction

Retinal hemorrhage (RH) is one of the most common ocular abnormalities in newborns and has a wide prevalence ranging from 2.6 to 50.0% [1–3]. The delivery mode, examination technique and time of examination after birth are considered as important factors that influence the rate of RH in newborns [4]. Vacuum extraction delivery was reportedly associated with the highest rate of RH, followed by spontaneous vaginal delivery and cesarean delivery [5,6]. Direct or indirect ophthalmoscopy is usually used for primary ocular screening of newborns. However, both techniques have some limitations. When performing direct ophthalmoscopy, some ocular abnormalities or RH in the peripheral regions may be missed due to the limited inspection range. Indirect ophthalmoscopy requires experienced and skilled experts for both performance and diagnosis, which may limit the widespread use and screening accuracy of this technique [7]. It was reported that birth-related hemorrhages may resolve quickly and can be of ‘small size and number’ [8], and those hemorrhages seen at 1 month of age were less likely birth-related [2]. But the other difference between NH and other hemorrhages found in other ocular or retinal disease was rarely reported.

In recent years, a wide-field digital imaging system (RetCam; Clarity Medical Systems, Pleasanton, CA, U.S.A.) has been applied with increasing frequency for regular ocular examination of newborns. This system can provide and record high-resolution, objective and wide-angle images of ocular diseases [4,9,10]. The system can provide a 130-degree field of view so that lesions in the peripheral regions, including RH, can be detected easily and quickly. Although the RetCam can provide quick and precise information regarding RH, there is a growing disparity between the increasing need for neonatal ocular examinations and the limited number of pediatric ophthalmologists. This problem is more severe in China, where there are more than 16 million newborns born each year. Therefore, an automated detection system for ocular abnormalities, including RH of different grades, based on fundus images of newborns is an urgent need.

Recent developments in deep learning techniques have provided evidence that artificial intelligence can help to diagnose ocular diseases, with an accuracy near or exceeding that attained by experienced experts, with much greater speed [11–15]. Gulshan et al. [11] developed a deep convolutional neural network (DCNN) to classify referable diabetic retinopathy using retinal fundus photographs with a sensitivity ranging from 87.0 to 97.5% and a specificity ranging from 93.4 to 98.5%. Similarly, Burlina et al. [12] applied a DCNN in the automated detection of age-related macular degeneration using color fundus images. Their DCNN yielded an accuracy of 88.4 to 91.6% with an area under the receiver operating characteristic curve (AUC) of 0.94 to 0.96, which are close to human performance levels.

The goal of the present study is to develop a DCNN model that can automatically determine the presence and severity of RH in newborns based on fundus images.

## Methods

### Data source

We used color fundus images of neonates from the National Science and Technology Basic Work program, a multi-center cross-sectional investigation involving the performance of fundus examinations by a wide-field digital imaging system (RetCam) on consecutive series of newborns with written informed consent. The digital images of the posterior pole and the superior, inferior, nasal and temporal retinal fields of each participant were captured and labeled by professional ophthalmologists. Neonatal RH was diagnosed and graded according to Egge’s classification [16]: (1) Grade 1: small retinal hemorrhage confined to the area around the optic nerve, associated with dot or fine linear bleeding; (2) Grade 2: slightly larger amount of retinal hemorrhage than Grade 1; patchy, dot, blot or flame-shaped hemorrhage, size does not exceed the optic disc diameter; (3) Grade 3: retinal hemorrhage more than the diameter of the optic disc area, a line of flame-shaped hemorrhage along vessels, macular hemorrhage The images of graded RH and normal fundi were used for training and testing of the DCNN. This research was approved by the ethics committee of National Research Institute for Family Planning.

### Model

**Figure 1:**
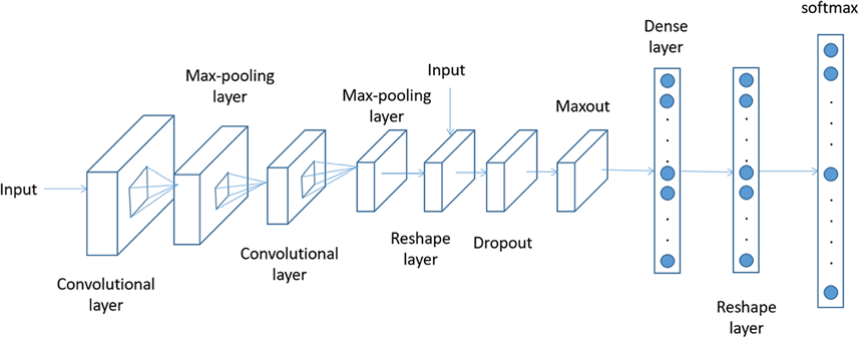
Framework of the deep convolution neural network

The architecture of the DCNN model is shown in Figure 1. The network comprised the following types of hidden layers:

- Convolutional layer (Conv): Performance of convolutional operation.
- Pooling layer (Max Pool): Performance of pooling operation.
- Maxout layer (Maxout): Use of a maxout activation function for non-linear changes, down-sampling.
- Dropout layer (Dropout): Prevention of over-fitting.
- Concatenate layer (Concat): Concatenation of input data.
- Dense layer (Dense): All nodes in the fully connected layer are connected to all nodes in the previous layer and are used to combine the features extracted from the front layers.
- Softmax layer (Softmax): Use of the softmax activation function to predict the results.

The details of the layers are shown in Table 1. All images were resized to 512 × 512, with three channels representing RGB. The image first went through the convolutional layer and max-pooling layer, and 7 × 7 feature maps of 256 channels were obtained. This part contained 16 convolutional layers and 5 max pool layers, similar to VGG19. The feature maps then went through the maxout layer, dropout layer and dense layer to improve the performance [17,18]. Finally, we applied the softmax function to predict the probability of each type (0, normal; 1, mild; 2, moderate; 3, severe) and used the type with the highest probability as the predicted result. We used Theano and Lasagna [19] to perform the DCNN model.

**Table 1:** Details of each layer in the convolution neural network

	Kernel	Strides	Number of filters	Feature map (height,width,channels)	Function
Conv	7*7	2*2	32	(256, 256, 32)	Extract features
Conv	5*5	2*2	32	(128, 128, 32)	
Max-pooling	3*3	2*2		(63, 63, 32)	Fuse feature and reduce feature map
Conv	3*3	1*1	32	(63, 63, 32)	
Conv	3*3	1*1	32	(63, 63, 32)	
Max-pooling	3*3	2*2		(31, 31, 32)	
Conv	3*3	1*1	64	(31, 31, 64)	
Conv	3*3	1*1	64	(31, 31, 64)	
Conv	3*3	1*1	64	(31, 31, 64)	
Conv	3*3	1*1	64	(31, 31, 64)	
Max-pooling	3*3	2*2		(15, 15, 64)	
Conv	3*3	1*1	128	(15, 15, 128)	
Conv	3*3	1*1	128	(15, 15, 128)	
Conv	3*3	1*1	128	(15, 15, 128)	
Conv	3*3	1*1	128	(15, 15, 128)	
Max-pooling	3*3	2*2		(7, 7, 128)	
Conv	3*3	1*1	256	(7, 7, 256)	
Conv	3*3	1*1	256	(7, 7, 256)	
Conv	3*3	1*1	256	(7, 7, 256)	
Conv	3*3	1*1	256	(7, 7, 256)	
Max-pooling	3*3	2*2		(3, 3, 256)	
Dropout				(3, 3, 256)	Prevent over fitting
Dense			1024		Purify features for classification
Maxout					
Dropout					
Dense			1024		
Maxout					
Dense			16		
Dense			8		
Softmax					classifier

### Data preparation

Our study included 48,996 fundus images collected from 3770 newborns (5–20 imagines for each one). All samples were carefully classified by experts as 765 normal fundi, 1023 grade 1 RH, 975 grade 2 RH and 1007 grade 3 RH. Each sample donated 5–20 eligible images after assessment in the present study. We divided each classification of newborns into a training set and testing set in a 7:3 ratio (Table 2).

**Table 2:** Summary of the training and testing dataset

Dataset	Number of images	Number of patients
Training		
Normal	6590	708
Grade 1 RH	7816	519
Grade 2 RH	11,470	685
Grade 3 RH	8210	727
Testing		
Normal	2762	299
Grade 1 RH	3678	246
Grade 2 RH	5130	290
Grade 3 RH	3340	296

Abbreviation: RH, retinal hemorrhage.

Because the external illumination conditions can be quite different when performing fundus examinations, all images went through a normalization step at the preprocessing stage to remove the illumination effect. For each pixel in the image, assuming the values on the three channels are *r, g* and *b*, the normalization changed the values on the three channels as follows:

$$\begin{array}{l} r^{\prime }=\frac{255\times r}{r+g+b}\\ g^{\prime }=\frac{255\times g}{r+g+b}\\ b^{\prime }=\frac{255\times b}{r+g+b} \end{array}$$

**Figure 2:**
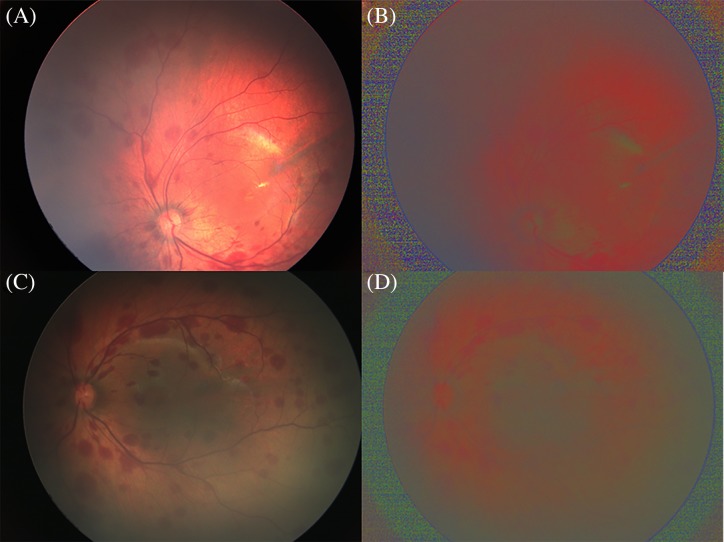
Normalization effect of digital fundus images (**A** and **C**) Fundus images taken under bright and dark illumination conditions. (**B** and **D**) Images after normalization.

Figure 2 shows the effect of normalizing the fundus images under different levels of illumination. After normalization (Figure 2B,D), the illumination effect was greatly reduced.

### Training

We adopted stochastic gradient descent and the Nesterov momentum method to train the model. The loss function was a combination of a continuous kappa loss together with the cross-entropy (or log) loss [20] as defined in (Copyright © 2015 Jeffrey De Fauw). We gradually decreased the learning rate to balance the learning speed and accuracy. The learning rate of each step is listed in Table 3 as the training procedure progressed. After 40,000 iterations of training, the loss and accuracy became stable.

**Table 3:** Change of learning rate as the training procedures went

Epochs	Learning rate
0–19	0.007
20–39	0.0035
40–59	0.0021
60–79	0.00105
80–99	0.0007
100–119	0.00035
120–139	0.00007
140–159	0.000035
160–179	0.000007
180–189	0.0000007
190–199	0.00000007

### Performance merit

We used the accuracy, sensitivity, specificity, positive predictive value (PPV) and negative predictive value (NPV) to assess the performance of the DCNN. Additionally, we applied a receiver operating characteristic (ROC) curve and computed the AUC. Notably, the detection of RH was a multiclass classification problem (normal, grade 1 RH, grade 2 RH and grade 3 RH). We transformed it into four binary classification problems for assessment and comparison of the performance levels: RH (yes vs. no), grade 1 RH (yes vs. no), grade 2 RH (yes vs. no) and grade 3 RH (yes vs. no). All statistical analyses were performed using SPSS version 19 (IBM Corp., Armonk, NY, U.S.A.).

## Results

**Figure 3:**
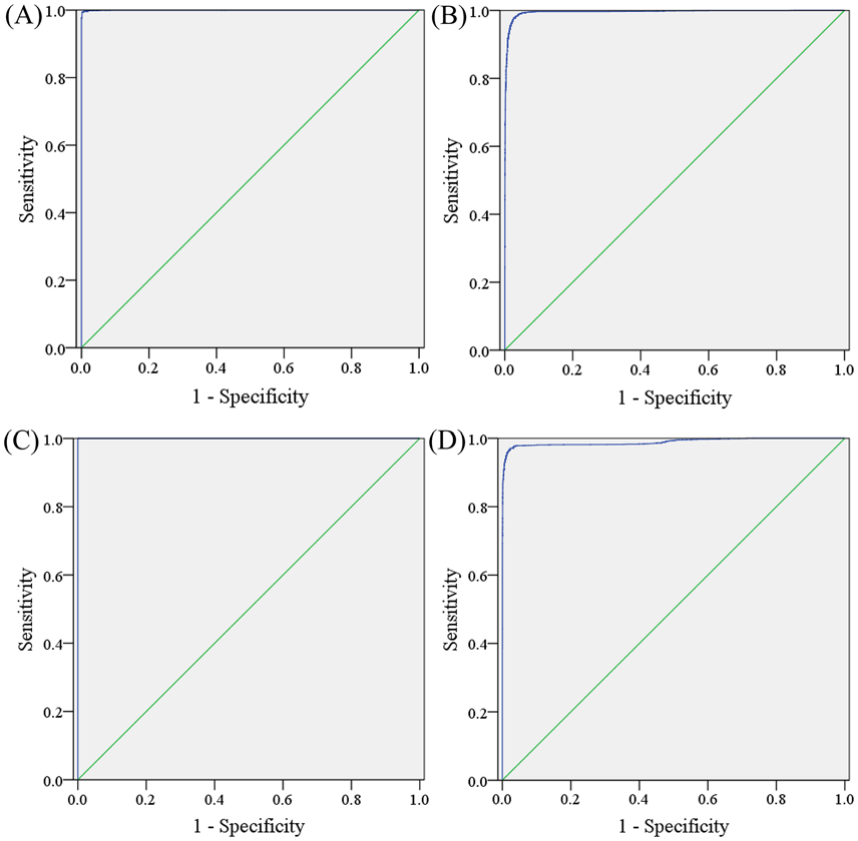
Receiver operating characteristic curve for performance of the neural network in the binary classification problem of retinal hemorrhage. (**A**) hemorrhage versus no hemorrhage, (**B**) grade 1 hemorrhage versus the others, (**C**) grade 2 hemorrhage versus the others and (**D**) grade 3 versus the others

We used 14,910 fundus images of 1131 newborns from the validation dataset to assess the performance of the trained DCNN. The performance levels of each classification are listed in Table 4, and the ROC curve of each classification is shown in Figure 3.

**Table 4:** Performance levels for machines experiments using fundus images dataset

Classification	Sensitivity	Specificity	PPV	NPV	Accuracy	AUC (95%CI)
RH	99.57	99.20	99.82	98.14	99.50	1 (0)
Grade 1 RH	97.85	97.85	93.62	99.30	97.85	0.995 (0.001)
Grade 2 RH	99.92	99.98	99.96	99.96	99.96	1 (0)
Grade 3 RH	93.11	99.26	97.31	98.04	97.88	0.989 (0.001)

Abbreviations: 95%CI, 95% confidence interval; AUC, area under ROC curve; NPV, negative predictive value; PPV, positive predictive value; RH, retinal hemorrhage. All values indicate percentages except for AUC.

The trained DCNN had high performance for prediction of RH regardless of severity, with a sensitivity of 99.57%, specificity of 99.20%, PPV predictive value of 99.82%, NPV of 98.14% and accuracy of 99.50%. The AUC was 1, indicating excellent performance of the DCNN for the diagnosis of RH.

The DCNN showed varying performance for classifying the severity of RH. The accuracy for grade 1, 2 and 3 RH was 97.85%, 99.96% and 97.88%, respectively. The AUC for each classification was 0.995, 1.000 and 0.989, respectively, implying that our DCNN has the best performance for classification of grade 2 RH. Overall, our model showed high performance levels in the detection and grading of RH using fundus images.

We also assessed the performance level of the DCNN on the multiclass classification problem (normal and grade 1, 2 or 3 RH). When the image classification obtained by the DCNN was in accordance with the original label, it was regarded as a correct classification. The overall accuracy was then calculated as the number of correct classifications divided by the total number of images. Finally, we obtained 14,528 correctly classified images, yielding an overall accuracy of 97.44%.

## Discussion

With the increasing understanding of the importance of neonatal ocular healthcare among parents, pediatric experts and ophthalmologists, there is a growing demand for early ocular examination worldwide, especially in developing countries. However, the numbers of ophthalmologists and individuals involved in ocular healthcare of newborns and children are still limited and are not increasing quickly enough to meet the rising demand. Thus, the development of automated and assistive systems for ocular screening is expected. The present study has demonstrated that artificial intelligence based on application of a DCNN can successfully classify and grade RH with high accuracy of 97.85 to 99.96% and an overall accuracy of 97.44%.

Compared with ophthalmoscopy or the red reflex test, wide-field digital imaging is the neonatal ocular screening method most widely recommended by the American Association of Pediatrics. This technique has some advantages in the comprehensive screening and documentation of neonatal ocular disorders. The system can be operated by a trained doctor or nurse with minimal clinical experience requirements. It also causes less distress in infants, increasing parents’ and ophthalmologists’ acceptance of this method for detection of RH and other disorders. The system can detect some fundus disorders other than RH, including retinopathy of prematurity [21–23], ocular birth defects [9], persistent hyperplastic primary vitreous [24], retinoblastoma [25] and others. Moreover, the digital system can generate high-resolution images of a wide range of the fundus that provide instant documentation of neonatal ocular disorders. The present study is the first to demonstrate that fundus images can also be used for automated detection of RH by combining these images with a DCNN.

RH is the most frequently encountered abnormality during neonatal universal ocular screening by the RetCam shuttle, accounting for more than 85% of all findings [9]. Most birth-related RH will spontaneously and quickly resolve because of its small size and number [8]. However, RH of great size, long duration or special location should be given closer attention because it can be a clinical sign of cerebral injury [4] or may affect the future development of vision. In the present study, we developed a DCNN that can successfully classify the severity of RH, which will support the long-term management of fundus hemorrhage.

In conclusion, this was a pilot exploration of the combination of artificial intelligence with digital fundus images for the detection of neonatal ocular abnormalities. We applied a DCNN in the classification and grading of RH, the most frequently encountered fundus abnormality in newborns, at high performance levels. In our next phase of development, we will further explore more DCNN models related to other ocular diseases or develop a comprehensive model for all the ocular diseases.

## Abbreviations

AUC: area under the receiver operating characteristic curve
DCNN: deep convolutional neural network
NH: neonatal hemorrhage
NPV: negative predictive value
PPV: positive predictive value
RH: retinal hemorrhage
ROC: receiver operating characteristic
